# Supplementary material for: Revealing intrinsic domains and fluctuations of moiré magnetism by a wide-field quantum microscope
Source: Nat Commun. 2023 Aug 29;14:5259. doi: 10.1038/s41467-023-40543-z (PMC10465594; doi:10.1038/s41467-023-40543-z)
Supplement: Supplementary file 1 — Supplementary Information [file 41467_2023_40543_MOESM1_ESM.pdf]

Supplementary Information for
Revealing intrinsic domains and fluctuations of moiré magnetism by a wide-field quantum microscope

Mengqi Huang[1,2,+], Zeliang Sun[3,+], Gerald Yan[1], Hongchao Xie[3], Nishkarsh Agarwal[4], Gaihua Ye[5], Suk Hyun Sung[4], Hanyi Lu[1], Jingcheng Zhou[1,2], Shaohua Yan[6], Shangjie Tian[6,7], Hechang Lei[6], Robert Hovden[4], Rui He[5], Hailong Wang[2,8], Liuyan Zhao[3,*], and Chunhui Rita Du[1,2,8,*]

[1]Department of Physics, University of California, San Diego, La Jolla, California 92093, USA
[2]School of Physics, Georgia Institute of Technology, Atlanta, Georgia 30332, USA
[3]Department of Physics, University of Michigan, Ann Arbor, Michigan 48109, USA
[4]Department of Materials Science and Engineering, University of Michigan, Ann Arbor, Michigan 48109, USA
[5]Department of Electrical and Computer Engineering, Texas Tech University, Lubbock, Texas 79409, USA
[6]Laboratory for Neutron Scattering, and Beijing Key Laboratory of Optoelectronic Functional Materials MicroNano Devices, Department of Physics, Renmin University of China, Beijing 100872, China
[7]School of Materials Science and Engineering, Anhui University, Hefei 230601, China
[8]Center for Memory and Recording Research, University of California, San Diego, La Jolla, California 92093

[*]Corresponding authors: lyzhao@umich.edu; c1du@physics.ucsd.edu
[+]These authors contributed equally

Supplementary Note 1. Transmission electron microscopy measurements of a low-twist-angle tDT CrI_3 device

We utilize diffraction-based transmission electron microscopy (TEM) measurements to characterize the microscopic lattice structure of low-twist-angle twisted-double-trilayer (tDT) CrI_3 encapsulated within hexagonal boron nitride nanoflakes. Thermo Fisher Talos, operated at 200kV and equipped with Gatan OneView camera, was employed for selected area electron diffraction (SAED) and dark-field (DF) TEM imaging. Supplementary Figure 1a shows third-order Bragg peaks ($\{3\bar{3}00\}$) from SAED pattern for a 0.3° tDT CrI_3 device. The local mean twist angle over a 860 nm × 860 nm sample area was measured to be $0.4° \pm 0.1°$ by fitting 2D gaussians to the Bragg peaks, giving a moiré period of ~100 nm. Note that the experimentally measured twist angle shows a small deviation (~0.1°) from the targeted value, within the accuracy of the stacking processes. The composite DF-TEM real space image from an ordered region of the specimen (Supplementary Fig. 1b)—obtained by averaging DF-TEM images from three third-order Bragg peaks 120° apart—features the superlattice structures with a periodicity commensurate with the moiré wavelength[1]. The observed distortion from the expected hexagonal lattice pattern is common for low-twist-angle tDT CrI_3 samples, which could be induced by lattice strain, relaxation, and local structural inhomogeneity[2].

Supplementary Note 2. Nitrogen-vacancy measurements of magnetic stray fields

A nitrogen-vacancy (NV) center is a point defect consisting of a substitutional nitrogen atom next to a carbon atom vacancy in a diamond crystal lattice[3,4]. The negatively charged NV center hosts an $S = 1$ electron spin that serves as a three-level quantum system[3,4]. Supplementary Fig. 2a shows the energy level diagrams for a single NV spin as a function of the magnetic field B_{NV} applied along the NV-axis. At $B_{NV} = 0$, the $m_s = \pm 1$ spin states are degenerate at an electron spin resonance (ESR) frequency of 2.87 GHz. As B_{NV} increases, the Zeeman coupling introduces an energy gap $2\tilde{\gamma}B_{NV}$ between the $m_s = -1$ and $m_s = +1$ NV spin states, which can be optically detected by measuring the spin-dependent NV photoluminescence (PL). $\tilde{\gamma}$ shown above denotes the gyromagnetic ratio of an NV spin.

The top panel of Supplementary Fig. 2b shows the optical and microwave sequence for pulsed ESR measurements. NV centers are first initialized to the $m_s = 0$ spin state by a microsecond-long green laser pulse followed by the application of a nanosecond-long microwave π-pulse to induce NV spin transitions. We sweep the frequency f of the microwave π pulses and measure the NV fluorescence by a charge-coupled device (CCD) camera during the application of the green laser readout pulse. When f matches the NV ESR frequencies, NV spins will be excited to the $m_s = \pm 1$ states, which are more likely to relax through a non-radiative pathway back to the $m_s = 0$ ground state and emit reduced PL[4]. The bottom panel of Supplementary Fig. 2b shows a representative NV ESR spectrum recorded at a single camera pixel over the CrI_3 sample at 10 K. In the current study, NV ensembles in the diamond substrates show four possible spin orientations with a mirror symmetry axis along the out-of-plane direction (z-axis)[5]. The angle between individual NV orientations and the z-axis is 54° (126°), and the applied external magnetic field B_{ext} is perpendicular to the sample plane. Considering the perpendicular anisotropy of CrI_3, the measured NV ESR spectrum shows four-fold degeneracy with only one pair of split NV spin

energy levels, from which the out-of-plane magnetic field B_z can be quantitatively obtained as follows[6]:

$$B_{\mathrm{NV}} = \pi(f_+ - f_-)/\tilde{\gamma} = B_z/\sqrt{3} \tag{1}$$

where $f_\pm$ denote the NV ESR frequencies corresponding to the $m_s = 0 \leftrightarrow \pm 1$ spin transitions. After subtracting the contribution of the external magnetic field B_{ext}, the out-of-plane component of stay field B_s emanating from CrI_3 samples can be obtained. By measuring the field-induced Zeeman splitting at every pixel of the captured image, we can have the B_s map over the entire field of view as shown in Figs. 2d-2g in the main text. In this work, we varied the measurement temperature in a broad range to reveal the stray field B_s response as shown in Supplementary Figs. 2c-2f.

Supplementary Note 3. Reconstruction of CrI_3 magnetization patterns

In this section, we present the detailed method to reconstruct magnetization patterns of CrI_3 samples from the measured stray field maps. Generally, the stray field distribution $\mathbf{B}_s(\mathbf{R})$ of a magnetic system is determined by its static magnetization distribution $\mathbf{M}(\mathbf{R}')$ as follows[7]:

$$\mathbf{B}_s(\mathbf{R}) = \int_\Omega d^3\mathbf{R}' \mathcal{D}(\mathbf{R},\mathbf{R}')\mathbf{M}(\mathbf{R}'), \tag{2}$$

where $\mathcal{D}(\mathbf{R},\mathbf{R}') = -\nabla_{\mathbf{R}}\nabla_{\mathbf{R}'}\left(1/|\mathbf{R}-\mathbf{R}'|\right)$ is the magnetostatic Green's function tensor between coordinates $\mathbf{R} = (x, y, z)$ and $\mathbf{R}' = (x', y', z')$, and Ω represents the CrI_3 sample space. Considering the atomically thin CrI_3 flakes used in the current study, we assume a uniform magnetization distribution along the thickness direction of the sample: $\mathbf{M}(\mathbf{R}') = \mathbf{M}(x', y')$. Also, we introduce the surface moment density $\boldsymbol{\sigma}(x',y') = \mathbf{M}(x',y')t_{\mathrm{M}}(x',y')$, where t_M is the thickness of individual atomic layers of CrI_3:

$$\mathbf{B}_s(\mathbf{R}) = \int_\Omega d^2\mathbf{r}' \mathcal{D}(\mathbf{R},\mathbf{R}')\boldsymbol{\sigma}(\mathbf{r}'), \tag{3}$$

where $\mathbf{r}' = (x', y')$. Note that we have neglected the variation of Green's function tensor along the thickness direction. Focusing on the magnetic field at the position of the NV centers ($z = d$), we take the Fourier transform in the x and y directions, where translational symmetries are present with $\mathbf{r} = (x, y)$ and $\mathbf{k} = (k_x, k_y)$:

$$\mathbf{B}_s(\mathbf{k}) = \int \mathbf{B}_s(\mathbf{r}, d)e^{i\mathbf{k}\cdot\mathbf{r}}d^2\mathbf{r},$$

$$\boldsymbol{\sigma}(\mathbf{k}) = \int \boldsymbol{\sigma}(\mathbf{r})e^{i\mathbf{k}\cdot\mathbf{r}}d^2\mathbf{r}. \tag{4}$$

Similarly, we can obtain the Green's function tensor in the Fourier space for $\alpha, \beta = x, y$[7]:

$$\begin{cases} D_{\alpha\beta}(\mathbf{k}) = -2\pi\left(k_\alpha k_\beta / k\right)e^{-kd}, \\ D_{\alpha z}(\mathbf{k}) = -2\pi i k_\alpha e^{-kd}, \\ D_{zz}(\mathbf{k}) = 2\pi k e^{-kd}, \end{cases} \tag{5}$$

where $k = |\mathbf{k}|$, thus

$$\mathbf{B}_{\mathrm{s}}(\mathbf{k}) = D(\mathbf{k})\boldsymbol{\sigma}(\mathbf{k}). \tag{6}$$

Due to the perpendicular anisotropy, CrI_3 magnetization is spontaneously aligned along the z-axis direction: $\boldsymbol{\sigma}(\mathbf{k}) = \sigma(\mathbf{k})\hat{\mathbf{z}}$. Here, we focus on the out-of-plane component of the stray field generated by the magnetic sample:

$$B_{\mathrm{s}}(\mathbf{k}) = 2\pi k e^{-kd}\sigma(\mathbf{k}), \tag{7}$$

Lastly, we introduce an inverse Fourier transform on Eq. (7) to reconstruct the CrI_3 magnetization patterns in the real space:

$$\sigma(\mathbf{r}) = \frac{1}{(2\pi)^2}\int \sigma(\mathbf{k})e^{-i\mathbf{k}\cdot\mathbf{r}}d^2\mathbf{k}. \tag{8}$$

Using the method presented above, we successfully reconstructed the magnetization patterns of tDT CrI_3 device A at different measurement temperatures as shown in Supplementary Figs. 3a-3h.

Supplementary Note 4. Theoretical simulations of stray fields generated by magnetic domains in tDT CrI_3

In this section, we qualitatively simulate the stray field patterns arising from the stacking induced (ferro)magnetic domains formed over arrays of moiré supercells in the ground state of low-twist-angle tDT CrI_3. Using the theoretical model proposed in Ref. 8, Supplementary Fig. 4a shows the characteristic hexagonal magnetic lattice structure of two moiré unit cells of tDT CrI_3 with opposite ferromagnetic order. The lattice constant of a moiré unit cell is estimated to be ~100 nm based on the targeted small twist angle of 0.3°. The white triangular region denotes the monoclinic (M) interlayer stacking geometry with antiferromagnetic (AFM) order while the remaining areas correspond to the rhombohedral (R) stacking geometry characterized by the ferromagnetic (FM) state[8]. Due to the prominent spatial magnetic "inhomogeneity" and suppressed inter-domain correlations, degeneracy of the ferromagnetic ground states could be lifted by external stimuli such as magnetic field, thermal cycles, and local defects, resulting in stacking-induced (ferro)magnetic domains formed over multiple moiré wavelength at a mesoscopic length scale. In the current simulations, we propose that individual moiré supercells with positive (red) and negative (blue) ferromagnetic order are randomly distributed over a lateral sample area of ~2 μm × 2 μm, as illustrated in Supplementary Fig. 4b. Using Eqs. (2)-(7), we are able to simulate the out-of-plane stray field B_s map on a plane with vertical distance d below the proposed magnetic pattern. Considering a diffraction limit of ~0.5 μm for wide-field measurements, we have applied a gaussian filter function to the simulation results to match our measurement resolution as presented in Supplementary Fig. 4c. One can see that the simulated stray field B_s map shows a spatially dependent distribution consisting of individual domains with opposite polarity on a micrometer length scale, in qualitative agreement with our experimental results.

Here, we would like to highlight that the "broad" field of view (~20 μm × 20 μm) enabled by our NV wide field techniques provides an excellent opportunity for investigating the extended moiré domain phases across multiple moiré periods in tDT CrI_3, which has not been revealed in

the previous study[8]. It is also instructive to note that our measurements were mostly performed under a small external magnetic field (tens of Gausses), which is favorable to the formation of spatially varying domain states in tDT CrI_3 especially in the magnetic phase transition regime.

Supplementary Note 5. NV measurements on a large-twist-angle tDT CrI_3 device

In this section, we show NV magnetometry results measured on tDT CrI_3 device B with a large twist angle of ~15° and reduced moiré wavelength of ~ 2.6 nm. Supplementary Fig. 5a shows an optical image of the prepared tDT CrI_3 device on a diamond substrate. Supplementary Fig. 5b shows the obtained magnetic stray field map measured at a temperature of 5 K with a perpendicular magnetic field B_{ext} of 71 G. In contrast with the multidomain feature observed in the low-twist-angle (~0.3°) tDT CrI_3 device A, device B shows a largely uniform magnetic domain over the twisted area, suggesting a pure magnetic order in the ground state. The reconstructed magnetization map is presented in Supplementary Fig. 5c. As reported in the previous studies, it is energetically unfavorable for large-twist-angle tDT CrI_3 to host coexisting antiferromagnetic and ferromagnetic orders[9]. Thus, interfacial ferromagnetic interaction is established between the two stacking CrI_3 trilayers, resulting in two layers of uncompensated magnetization. It is worth noting that the reconstructed magnetization in the twisted area shown in Supplementary Fig. 5c is approximately twice of that measured for pristine CrI_3 trilayer, in consistent with the picture discussed above.

Supplementary Note 6. NV relaxometry measurements of tDT CrI_3

In this work, we use NV relaxometry to detect the longitudinal spin noise arising from tDT CrI_3 samples and to investigate their spin fluctuations as a function of temperature and frequency (discussed in the following section). The top panel of Supplementary Fig. 6a shows the measurement protocol for NV relaxometry. NV centers are first initialized to the $m_s = 0$ spin state with a green laser pulse. Spin fluctuations in tDT CrI_3 will generate fluctuating magnetic fields, which could couple to proximate NV centers via dipole-dipole interactions[10–12]. The fluctuating magnetic fields at the NV ESR frequencies will induce $m_s = 0 \leftrightarrow \pm 1$ spin transitions, leading to an enhancement of NV spin relaxation rate Γ at the corresponding NV ESR frequencies. After a delay time t, a microwave π-pulse at the NV ESR frequency is applied and the corresponding NV spin state occupations are readout by a second green laser pulse. NV spin relaxation rate Γ can be quantitatively obtained by fitting the integrated NV PL with an exponential decay function PL(t) = $A_0 + Ae^{-t\Gamma}$, where A_0 and A are constants[11]. Note that Γ includes two components Γ_0 and Γ_M corresponding to the intrinsic and magnetic fluctuation induced NV relaxations, respectively. The intrinsic component Γ_0 can be measured on NV centers in a diamond area not being covered by the CrI_3 device as illustrated in Supplementary Fig. 6a. Γ_M can be obtained by performing NV relaxation measurements on NV centers underneath the CrI_3 device (Supplementary Fig. 6b).

Supplementary Fig. 6c shows a set of representative NV spin relaxation spectra for the $m_s = 0 \leftrightarrow -1$ spin transition measured on a single camera pixel located on the bare diamond substrate. The external magnetic field is 71 G applied along the out-of-plane direction and the NV ESR frequency is 2.78 GHz. The intrinsic NV spin relaxation rate Γ_0 is obtained to be 4.0 kHz, 3.8 kHz, 4.1 kHz, and 4.2 kHz at 36 K, 47 K, 57 K, and 92 K, respectively. Supplementary Fig. 6d shows NV spin relaxation spectra measured on a single camera pixel over the tDT CrI_3 sample area at the

same ESR frequency and temperature. Subtracting the intrinsic contribution Γ_0, spin fluctuation induced NV relaxation $\Gamma_M = \Gamma - \Gamma_0$ can be quantiatively obtained. By performing wide-field imaging measurements over the entire sample area, Figs. 4a − 4g in the manuscript present a series of Γ_M maps of the tDT CrI_3 device A measured at different temperatures.

Supplementary Note 7. Inferring intrinsic spin diffusion constant and longitudinal magnetic susceptibility of tDT CrI_3

In this section, we provide the details for extracting the intrinsic spin diffusion constant and static longitudinal magnetic susceptibility of tDT CrI_3 from NV relaxometry results. Supplementary Fig. 7a shows the optical image of a low-twist-angle (~0.3°) tDT CrI_3 device C transferred onto a [111] oriented single crystalline diamond membrane for relevant measurements. NV ensemble is implanted on the surface of the [111] diamond for wide-field magnetometry measurements[13]. Due to the strong perpendicular magnetic anisotropy, the magnon band gap of CrI_3 is larger than the NV ESR frequency under the field conditions of our measurements, thus, the measured NV spin relaxation rate $\Gamma_M(f_{ESR})$ is mainly driven by the longitudinal spin fluctuations in CrI_3, which can be described using a two-magnon noise model[10,12,14]:

$$\Gamma_M(f_{ESR}) = \frac{1}{2\pi f_{ESR}\beta} G(\theta) \int_{-(t_M+d)}^{-d} dz \int_0^{\infty} dk k^3 e^{2kz} \chi''\left(k, f_{ESR}\right), \tag{9}$$

where $\beta = 1/k_B T$, k_B is the Boltzmann constant, d is vertical distance between NV centers and the twisted CrI_3 sample, t_M is the thickness of the magnetic device, T is the temperature, and $\chi''(k, f_{ESR})$ is the imaginary part of the dynamical longitudinal spin susceptibility. The geometric factor $G(\theta)$ can be expressed as:

$$G(\theta) = \frac{(\gamma\tilde{\gamma})^2 \pi}{2}(5 - \cos 2\theta), \tag{10}$$

where $\tilde{\gamma}$ and γ are the gyromagnetic ratio of NV centers and CrI_3, respectively, and $\theta = 0°$ is the angle between the NV spin orientation and the magnetic easy axis of CrI_3. Note that the effective NV axis is along the out-of-plane direction for [111] oriented diamond, allowing for application of an external magnetic field in a relatively large range. To obtain the expression of dynamical longitudinal spin susceptibility $\chi''(k, f_{ESR})$, we describe the magnetic system studied by a diffusion equation. Assuming U(1) symmetry, the diffusion equation for spins oriented along the direction of the magnetic order parameter s^z can be written as[12,15]:

$$\partial_t s^z + \nabla \cdot \mathbf{j}_s = -\frac{1}{\tau_s}\left(s^z - \chi H\right). \tag{11}$$

Here, we have introduced the spin-relaxation time τ_s and the spin current $\mathbf{j}_s = -\sigma \nabla \mu$, where σ is the spin conductivity, $\mu = \chi^{-1} s^z - H$ is the spin chemical potential, χ is the static uniform longitudinal spin susceptibility, and H is an external perturbation thermodynamically conjugate to the spin density. By introducing the intrinsic spin diffusion coefficient $D = \sigma / \chi$, we have:

$$\partial_t s^z - D\nabla^2 s^z + D\chi\nabla^2 H = -\frac{1}{\tau_s}\left(s^z - \chi H\right). \tag{12}$$

By introducing the Fourier transform $s^z\left(\mathbf{k}, f_{\text{ESR}}\right) = \int e^{i(\mathbf{k}\cdot\mathbf{r}-2\pi ft)} s^z\left(\mathbf{r},t\right) d^2\mathbf{r}dt$, we have:

$$s^z\left(\mathbf{k}, f_{\text{ESR}}\right) = \frac{\chi\left(Dk^2 + 1/\tau_s\right)}{2\pi i f_{\text{ESR}} + \left(Dk^2 + 1/\tau_s\right)} H. \tag{13}$$

Thus, the imaginary part of the dynamical longitudinal spin susceptibility can be written as:

$$\chi''\left(k, f_{\text{ESR}}\right) = \frac{2\pi\chi\left(Dk^2 + 1/\tau_s\right) f_{\text{ESR}}}{\left(2\pi f_{\text{ESR}}\right)^2 + \left(Dk^2 + 1/\tau_s\right)^2}. \tag{14}$$

Combining Eq. (9) and Eq. (14), and considering the limit of a long relaxation time ($\tau_s \to \infty$), the spin relaxation rate of an NV center driven by longitudinal spin fluctuations can be expressed as:

$$\Gamma_{\text{M}}(f_{\text{ESR}}) = \frac{G(\theta)}{2\beta}\int dk k^2 e^{-2kd}\left(1 - e^{-2kt_M}\right)\frac{\chi D k^2}{\left(2\pi f_{\text{ESR}}\right)^2 + \left(Dk^2\right)^2}. \tag{15}$$

Note that we have introduced the magnetic longitudinal susceptibility $\chi_0 = \gamma^2\chi$ in order to express the susceptibility in the conventional unit of emu·cm^{-3}·Oe^{-1}.

Supplementary Fig. 7b shows NV spin relaxation rate Γ_{M} measured on the tDT CrI_3 device C as a function of the ESR frequency f_{ESR} at 40 K. Here, f_{ESR} is systematically changed by varying the magnitude of the external perpendicular magnetic field. By fitting the results using Eq. (15), the intrinsic spin diffusion constant D and static magnetic longitudinal susceptibility χ_0 of the tDT CrI_3 sample are obtained to be (4.2 ± 0.3) ×10^{-5} m^2/s and (4.0 ± 0.2) ×10^{-2} emu·cm^{-3}·Oe^{-1} at 40 K, respectively.

Supplementary Note 8. Extended NV wide-field magnetometry measurements of tDT CrI_3 device D

We have performed extended NV wide-field magnetometry measurements on tDT CrI_3 device D to further confirm the reproducibility of our presented results. Supplementary Figs. 8a and 8b show optical images of an original 3-layer CrI_3 flake and the prepared twisted device, respectively. The thick black dashed lines (Supplementary Fig. 8a) denote the position where the flake was torn for twisted stacking, and the tDT sample with a targeted twist angle of 0.3° was positioned on a diamond substrate for wide-field magnetometry measurements. In general, we have observed similar experimental features as reported in the main text. Supplementary Fig. 8c presents the measured stray field map of the tDT CrI_3 sample after field-cooling with an out-of-plane external magnetic field of ~71 G, showing coexisting (ferro)magnetic domains with opposite polarity. By reversing the polarity of the magnetic cooling field, the sign of individual domains of tDT CrI_3 changes accordingly (Supplementary Fig. 8d). Supplementary Figs. 8e and 8f show the corresponding magnetization maps of the tDT CrI_3 sample with positive and negative field cooling.

Supplementary Figs. 9a-9e present the stray field maps of tDT CrI_3 device D with application of an external magnetic field of 71 G at varying temperatures from 5 K to 62 K. The

magnitude of the stray field emanating from the magnetic device is found to decrease as increasing temperature and eventually reaches zero above the Curie temperature. Supplementary Fig. 9f tracks the temperature dependence of the stray field B_s measured at two local points with opposite domain polarity. Below the magnetic transition temperature, we observed a monotonic decrease of the magnitude of the measured stray field due to the reducing CrI_3 magnetization. Notably, B_s decays to zero around the Curie temperature, indicating demagnetization of the sample.

Supplementary Figs. 10a-10e show the temperature dependence of the NV relaxation rate Γ_M maps measured for the tDT CrI_3 sample D from 21 K to 92 K. The external magnetic field of 71 G is applied along the out-of-plane direction of the sample. The measured relaxation rate maps exhibit a high spatial uniformity over the tDT CrI_3 area, consistent with the results presented in the main text. The measured NV spin relaxation rate Γ_M shows a suppressed value in the low temperature regime (Supplementary Fig. 10a). As the temperature increases, Γ_M reaches a maximum value around the magnetic transition temperature due to an increase of the magnetic susceptibility (Supplementary Fig. 10d). Supplementary Fig. 10f plots the temperature dependence of Γ_M measured in the tDT CrI_3 sample area, showing a distinct peak value of 9.8 kHz around ~52 K.

References

1. Xie, H. *et al.* Evidence of non-collinear spin texture in magnetic moiré superlattices. *Nat. Phys.* (2023). https://doi.org/10.1038/s41567-023-02061-z
2. Sung, S. H. *et al.* Torsional periodic lattice distortions and diffraction of twisted 2D materials. *Nat Commun* **13**, 7826 (2022).
3. Doherty, M. W. *et al.* The nitrogen-vacancy colour centre in diamond. *Physics Reports* **528**, 1–45 (2013).
4. Rondin, L. *et al.* Magnetometry with nitrogen-vacancy defects in diamond. *Rep. Prog. Phys.* **77**, 056503 (2014).
5. Doherty, M. W. *et al.* Measuring the defect structure orientation of a single NV^- centre in diamond. *New J. Phys.* **16**, 063067 (2014).
6. Chen, H. *et al.* Revealing room temperature ferromagnetism in exfoliated Fe_5GeTe_2 flakes with quantum magnetic imaging. *2D Mater.* **9**, 025017 (2022).
7. Guslienko, K. Y. & Slavin, A. N. Magnetostatic Green's functions for the description of spin waves in finite rectangular magnetic dots and stripes. *Journal of Magnetism and Magnetic Materials* **323**, 2418–2424 (2011).
8. Song, T. *et al.* Direct visualization of magnetic domains and moiré magnetism in twisted 2D magnets. *Science* **374**, 1140–1144 (2021).
9. Xu, Y. *et al.* Coexisting ferromagnetic–antiferromagnetic state in twisted bilayer CrI_3. *Nat. Nanotechnol.* **17**, 143–147 (2022).
10. van der Sar, T., Casola, F., Walsworth, R. & Yacoby, A. Nanometre-scale probing of spin waves using single electron spins. *Nat Commun* **6**, 7886 (2015).
11. Du, C. *et al.* Control and local measurement of the spin chemical potential in a magnetic insulator. *Science* **357**, 195–198 (2017).
12. Flebus, B. & Tserkovnyak, Y. Quantum-impurity relaxometry of magnetization dynamics. *Physical Review Letters* **121**, 187204 (2018).
13. McLaughlin, N. J. *et al.* Quantum imaging of magnetic phase transitions and spin fluctuations in intrinsic magnetic topological nanoflakes. *Nano Lett.* **22**, 5810–5817 (2022).
14. Wang, H. *et al.* Noninvasive measurements of spin transport properties of an antiferromagnetic insulator. *Sci. Adv.* **8**, eabg8562 (2022).
15. Fang, H., Zhang, S. & Tserkovnyak, Y. A generalized model of magnon kinetics and subgap magnetic noise. *Phys. Rev. B* **105**, 184406 (2022).

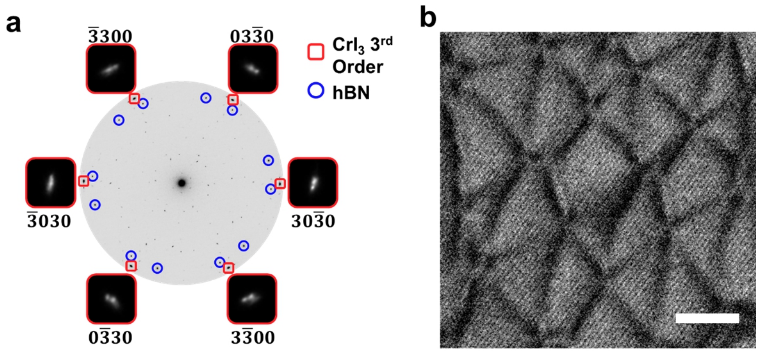

Supplementary Fig. 1. a SAED patterns of the third-order Bragg peaks of a low-twist-angle tDT CrI_3 device (red rectangles) from a surveyed area. **b** Averaged DF-TEM real space image constructed from $[3\bar{3}00]$, $[\bar{3}030]$ and $[03\bar{3}0]$ Bragg peaks showing the characteristic hexagonal superlattice structure of the tDT CrI_3 device. The scale bar is 30 nm.

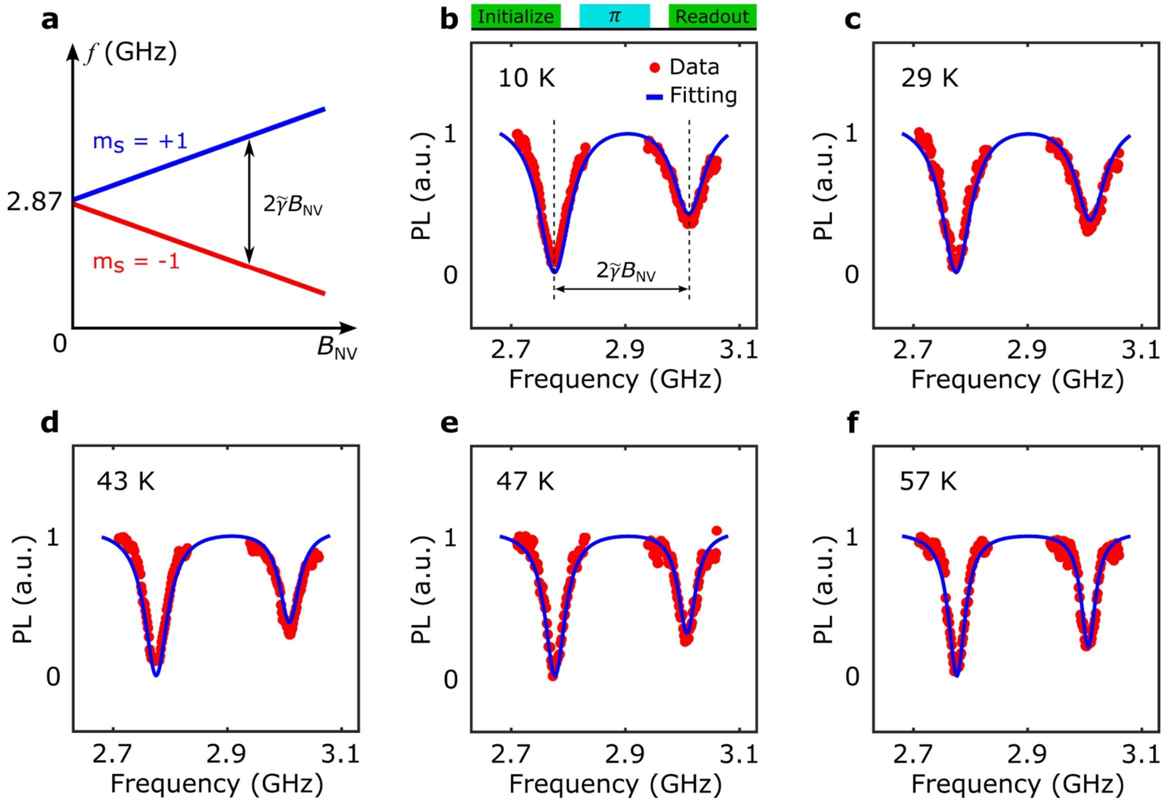

Supplementary Fig. 2. NV ESR spectra measured at different temperatures. a Zeeman splitting of NV spin energy level in the presence of an external magnetic field B_{NV} along the NV axis. **b** Top panel: the optical and microwave sequence used for pulsed NV ESR measurements. Bottom panel: ESR spectrum measured on NV centers underneath a tDT CrI_3 device at a temperature of 10 K. **c-f** NV ESR spectra measured at other temperatures of 29 K **(c)**, 43 K **(d)**, 47 K **(e)**, 57 K **(f)**. Red points and the blue lines correspond to the experimental data and fitting curves, respectively.

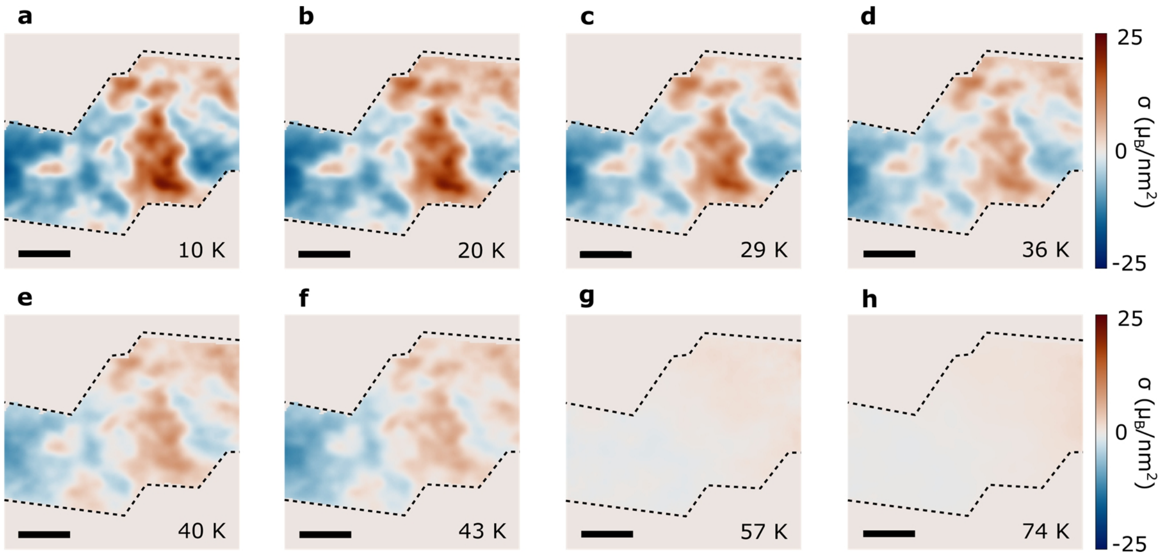

Supplementary Fig. 3. Temperature dependence of the tDT CrI_3 magnetization. a-h Reconstructed magnetization maps of the tDT CrI_3 device A at temperatures of 10 K (**a**), 20 K (**b**), 29 K (**c**), 36 K (**d**), 40 K (**e**), 43 K (**f**), 57 K (**g**) and 74 K (**h**). The black dashed lines outline the boundary of the tDT CrI_3 device, and the scale bars are 3 μm.

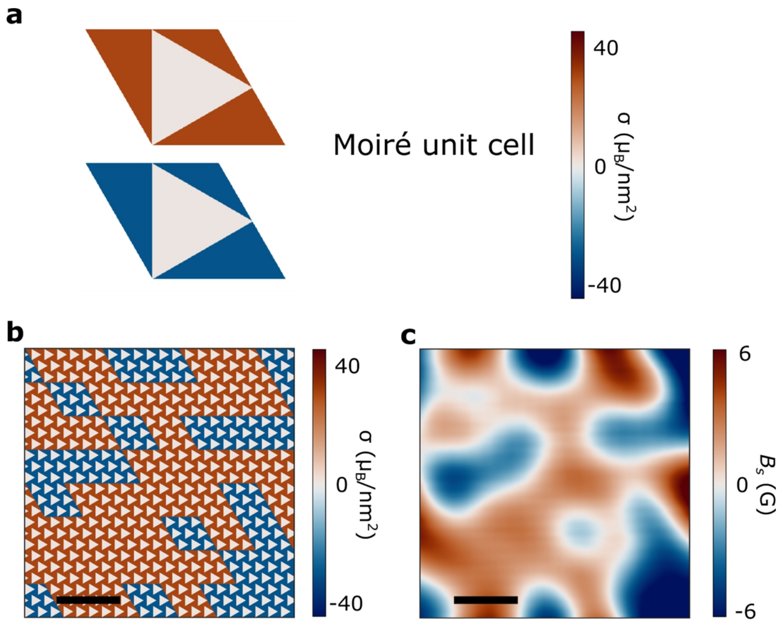

Supplementary Fig. 4. Theoretical simulations of magnetic domain features of low-twist-angle tDT CrI_3. a Schematic of the magnetization structure of moiré unit cells with positive (up) and negative (bottom) ferromagnetic ordering. The triangular region denotes the antiferromagnetic (AFM) order and the remaining areas correspond to the ferromagnetic state. **b** Proposed magnetization pattern of low-twist-angle tDT CrI_3 for theoretical simulations. Individual moiré unit cells with positive (red) and negative (blue) ferromagnetic order are randomly distributed over a lateral sample area of 2 μm × 2 μm. The lateral length of individual moiré cells is 100 nm. **c** Simulated stray field map arising from the proposed magnetization pattern. Scale bar is 500 nm in Fig. **b** and **c**.

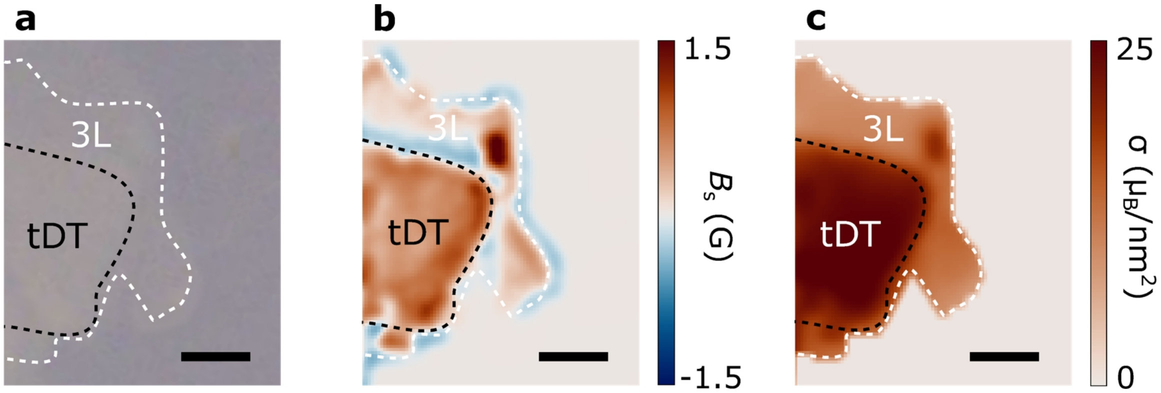

Supplementary Fig. 5. NV wide-field imaging of a large-twist-angle tDT CrI_3 device. a Optical image of the prepared tDT CrI_3 device B with a targeted twist angle of 15°. **b** An NV stray field map of tDT CrI_3 device B. **c** Reconstructed magnetization map showing a uniform magnetic distribution over the tDT and pristine trilayer regions. The black and white dashed lines in the figures mark the boundaries of two CrI_3 trilayer flakes, and the scale bar is 2 μm.

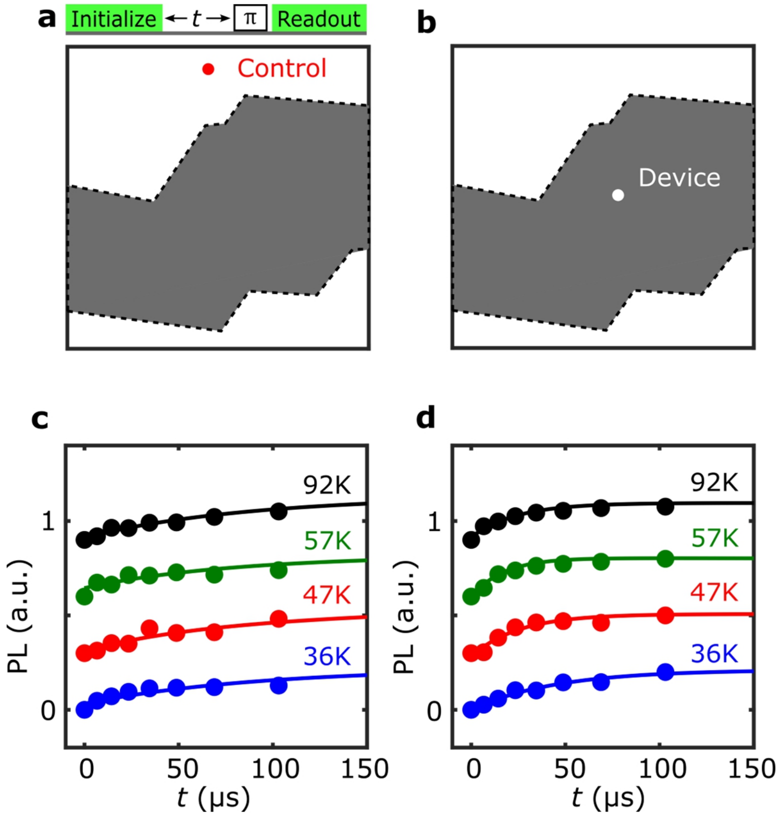

Supplementary Fig. 6. NV relaxometry measurements. **a** Top panel: optical and microwave measurement sequence for NV relaxometry measurements. Bottom panel: control experiment on NV centers in the diamond area that is not covered with the CrI_3 device. **b** NV relaxometry measurements on NV centers underneath the CrI_3 device. **c, d** Four sets of NV relaxometry raw data showing NV (PL) of the $m_s = -1$ spin state as a function of the delay time *t* at temperatures of 36 K, 47 K, 57 K, and 92 K. Results presented in Figs. **c** and **d** correspond to the measurement geometries shown in Figs. **a** and **b**, respectively. Experimental spectra are vertically offset for visual clarity.

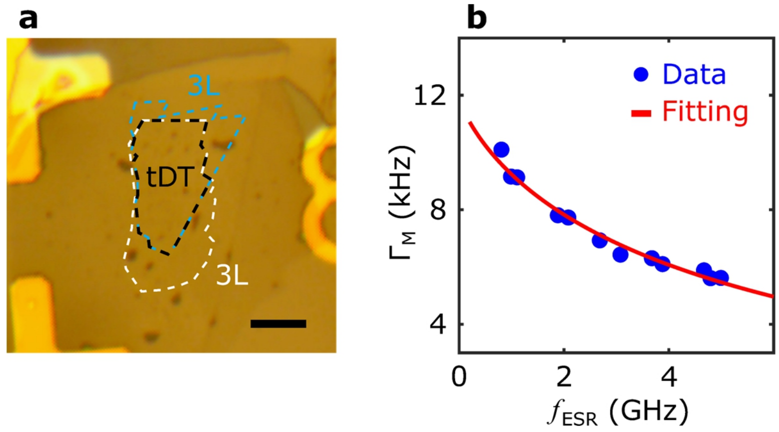

Supplementary Fig. 7. Extraction of intrinsic spin diffusion constant and longitudinal susceptibility of tDT CrI_3. a Optical image of tDT CrI_3 device C for frequency dependent NV spin relaxometry measurements. The white and blue dashed lines mark the boundaries of two 3-layer CrI_3 flakes, and the twisted area is outlined by the black dashed lines. The scale bar is 15 μm. **b** Spin fluctuation induced NV spin relaxation rate Γ_M (blue dots) measured as a function of the NV ESR frequency f_{ESR} at 40 K. The experimental results can be fitted to a theoretical model (red curve), from which the intrinsic spin diffusion constant and longitudinal susceptibility of the magnetic sample can be extracted.

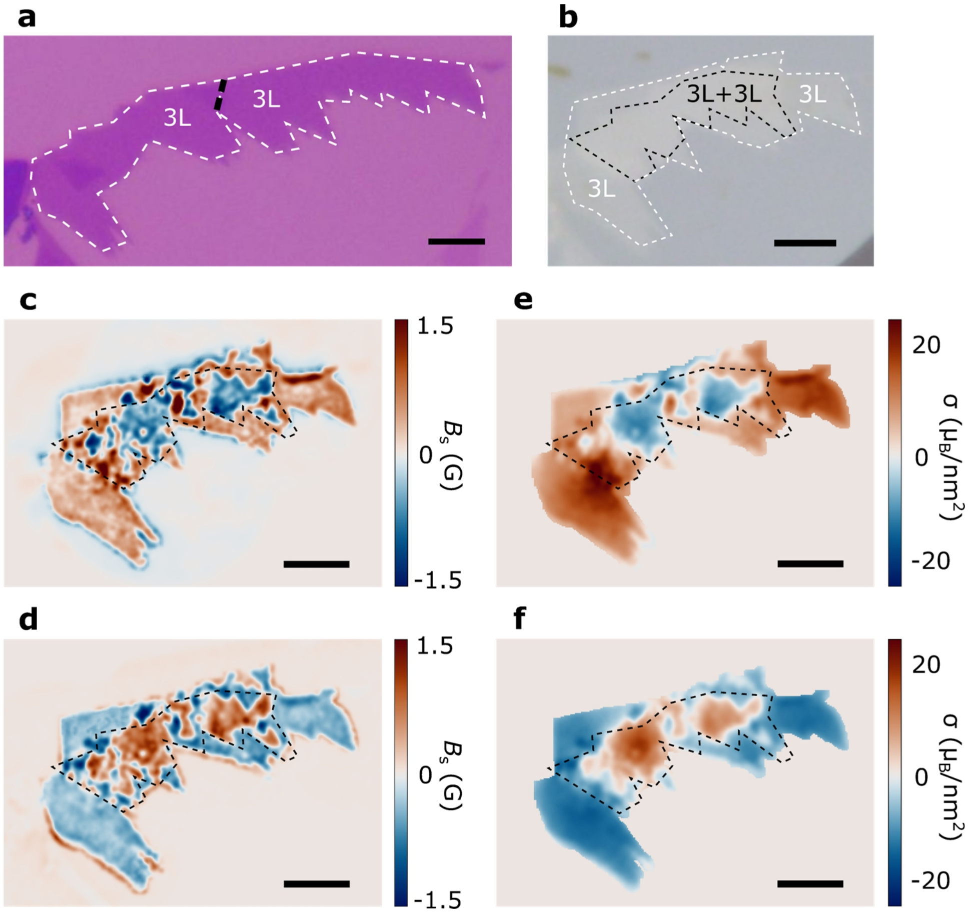

Supplementary Fig. 8. Extended NV wide-field magnetometry measurements of tDT CrI_3 device D. a, **b** Optical images of an original 3-layer CrI_3 flake and the prepared tDT CrI_3 device D. The white dashed lines mark the boundary of the 3-layer CrI_3 flake, and the thick black dashed line indicates the position where the flake was torn into two pieces for twisted stacking (**a**). The twisted area is outlined by the thin black dashed lines. **c, d** Stray field maps of the CrI_3 sample after field-cooling with a positive (71 G) and negative (− 71 G) perpendicular field at 5 K. **e, f** Reconstructed magnetization patterns of the CrI_3 sample after field-cooling with a positive and negative perpendicular field of at 5 K. All scale bars are 5 μm.

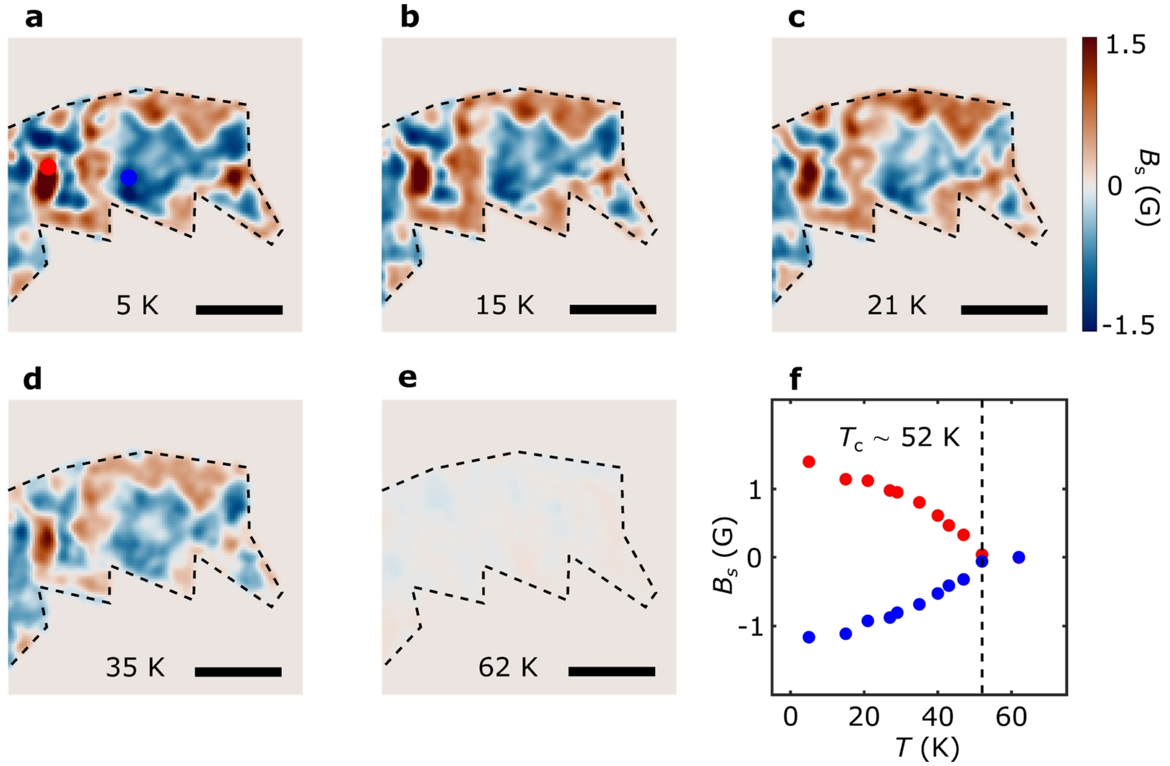

Supplementary Fig. 9. Temperature dependence of stray field maps of tDT CrI_3 device D. a-e Stray field maps of tDT CrI_3 device D measured at temperatures of 5 K (**a**), 15 K (**b**), 21 K (**c**), 35 K (**d**), and 62 K (**e**). The black dashed lines outline the area of tDT CrI_3 sample, and the scale bar is 3 μm. **f** Temperature dependence of magnetic stray field B_s measured at two local sample areas with opposite domain polarity (blue and red points shown in Fig. **a**).

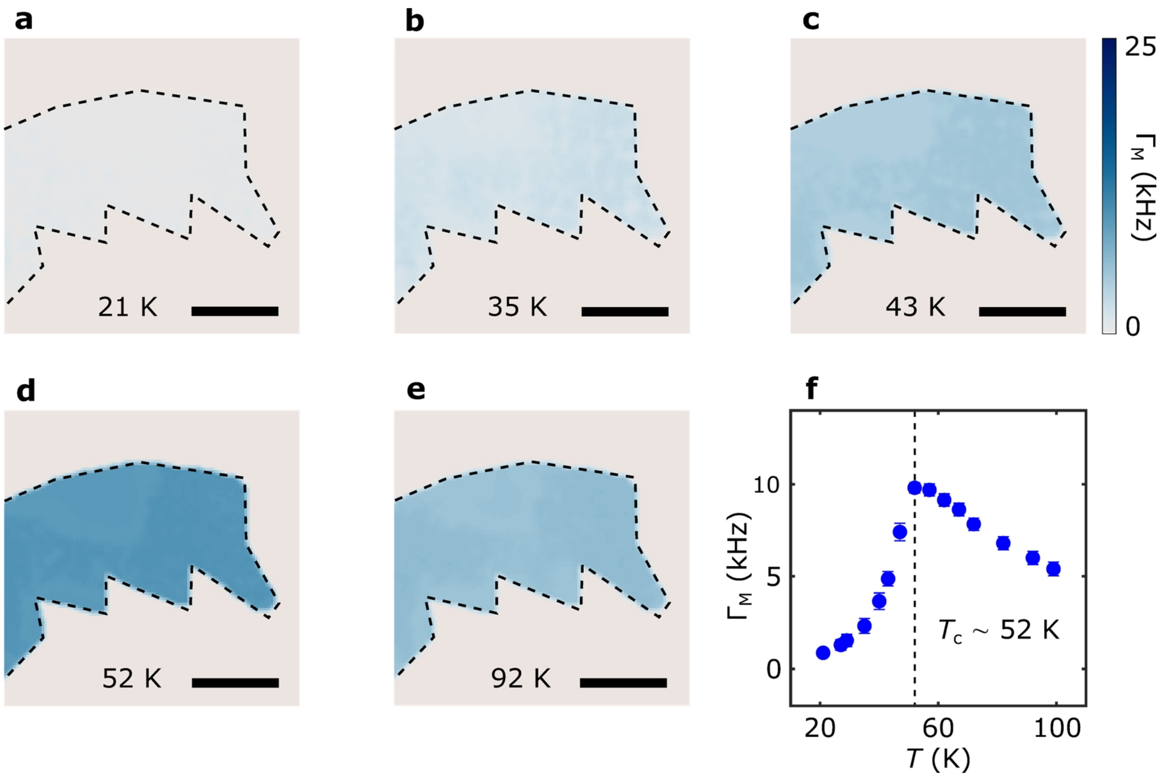

Supplementary Fig. 10. Temperature dependence of NV spin relaxation maps of tDT CrI_3 device D. a-e NV spin relaxation maps of tDT CrI_3 device D measured at temperatures of 21 K (**a**), 35 K (**b**), 43 K (**c**), 52 K (**c**), and 92 K, respectively. The black dashed lines outline the area of the tDT CrI_3 sample, and the scale bar is 3 μm. **f** Temperature dependence of the NV spin relaxation rate Γ_M measured on the tDT CrI_3 sample, from which the magnetic phase transition temperature of the sample is estimated to be ~52 K.
